# Hepatic Subcapsular Flow as a Significant Diagnostic Marker for Biliary Atresia: A Meta-Analysis

**DOI:** 10.1155/2020/5262565

**Published:** 2020-03-01

**Authors:** Chao Sun, Bin Wu, Jiang Pan, Lulu Chen, Wenxian Zhi, Ruze Tang, Dongliang Zhao, Wanliang Guo, Jian Wang, Shungen Huang

**Affiliations:** ^1^Department of General Surgery, Soochow University Affiliated Children's Hospital, Suzhou, China; ^2^Radiology Department, Soochow University Affiliated Children's Hospital, Suzhou, China

## Abstract

**Aim:**

Increasing evidence indicates that hepatic subcapsular flow (HSF) can serve as a noninvasive ultrasonographic marker for the early diagnosis of biliary atresia (BA). However, results regarding its diagnostic accuracy are inconsistent and inconclusive. We conducted this meta-analysis with an aim to systematically evaluate the diagnostic value of HSF in predicting BA.

**Methods:**

A comprehensive literature search of four databases was conducted to identify the eligible studies. All analyses were performed using STATA 12.0.

**Results:**

Nine studies from eight articles containing 368 patients and 469 controls were included in our meta-analysis. Briefly, the values for pooled sensitivity, specificity, positive likelihood ratio (PLR), negative likelihood ratio (NLR), diagnostic odds ratio (DOR), and area under the curve (AUC) were 0.95 (95% CI 0.88-0.98), 0.92 (95% CI 0.85-0.96), 11.6 (95% CI 6.3-21.5), 0.06 (95% CI 0.02-0.14), 201 (95% CI 59-689), and 0.98 (95% CI 0.96-0.99), respectively. Additionally, metaregression along with subgroup analysis based on various covariates revealed the potential sources of heterogeneity and the detailed diagnostic value in each subgroup.

**Conclusion:**

Our meta-analysis showed that HSF assay could provide high accuracy in predicting BA patients and non-BA individuals. However, further studies with better design and larger sample size are required to support the results of the present study.

## 1. Introduction

Biliary atresia (BA), a rare neonatal cholangiopathy of unknown etiology, can rapidly progress to biliary cirrhosis and early death [1]. The current mainstay treatment for BA is the Kasai portoenterostomy, whose prognosis is found to be related to the timing of surgical intervention [2, 3]. If patients are identified at an early age, most of them can achieve long-term survival [4]. However, it is very difficult to differentiate BA cases from non-BA cases, especially within the first 60 days of life, and thus, many patients cannot receive the optimal surgical treatment of Kasai portoenterostomy [5, 6]. Thus, there is an urgent need for diagnosis at an early age.

The early diagnosis of BA is a global problem. Due to commonalities in clinical features of extrahepatic BA and intrahepatic cholestasis, differentiating BA from other causes of neonatal cholestasis, such as idiopathic neonatal hepatitis, often presents a clinical challenge [7, 8]. Currently, some progress has been made in the diagnosis of BA, including stool color card screening [9], ultrasonography [10], magnetic resonance cholangiopancreatography [11], radionuclide hepatobiliary scanning [12], and endoscopic retrograde cholangiopancreatography [13]. However, patients may occasionally lack the symbolic signature for the early diagnosis of BA; thus, many of them may miss the optimal timing of surgical treatment. Intraoperative cholangiography and liver biopsy remain the gold standard for diagnosing BA, but they are invasive examination methods for children [14–16]. Although some markers and examinations have been proved distinct and have the potential for diagnosis of BA, an individual, ideal method has not yet been acknowledged.

Ultrasonography, a noninvasive, cost-efficient, and simple primary method, can clearly show the structure of porta hepatis (sonographic triangular cord sign, gallbladder, hepatic arterial diameter, and ultrasonic hepatic subcapsular flow (HSF)); thus, it may potentially be the preferred method for the early diagnosis of BA and exclusion of BA [17]. However, the diagnostic performances of the gallbladder and hepatic arterial diameter are not satisfactory due to the low sensitivity or low specificity [18]. Recent studies have focused on the clinical value of HSF in the early diagnosis of BA [19–21]. HSF, which has been confirmed to correlate with mild fibrosis and inflammation of the liver, extends to the hepatic surface in all patients with BA, thus indicating that it could be a novel potential marker [20]. But its diagnostic accuracy remains inconsistent. For example, in Lee et al.'s study, the sensitivity and specificity were 100% and 86%, respectively [22], while in El-Guindi et al.'s study, the sensitivity and specificity were 96.3% each [21]. These findings compelled us to carry out comprehensive research to precisely evaluate the diagnostic accuracy of HSF.

## 2. Materials and Methods

### 2.1. Literature Search Strategies

This meta-analysis was based on the principles of the Preferred Reporting Items in Systematic Reviews and Meta-Analysis (PRISMA) statement [23]. A literature search was conducted to identify the relevant records for HSF in the diagnosis of BA in databases including PubMed, EMBASE, Chinese National Knowledge Infrastructure (CNKI), and Technology of Chongqing (VIP) up to January 1, 2019. The following keywords were used in this search: (((((((((((Biliary Atresia[Title/Abstract]) OR Atresia, Biliary[Title/Abstract]) OR Biliary Atresia, Extrahepatic[Title/Abstract]) OR Biliary Atresias, Extrahepatic[Title/Abstract]) OR Atresias, Extrahepatic Biliary[Title/Abstract]) OR Atresia, Extrahepatic Biliary[Title/Abstract]) OR Extrahepatic Biliary Atresia[Title/Abstract]) OR Extrahepatic Biliary Atresias[Title/Abstract]) OR Familial Extrahepatic Biliary Atresia[Title/Abstract]) OR Idiopathic Extrahepatic Biliary Atresia[Title/Abstract]) OR “Biliary Atresia”[Mesh])). AND (((((((((((((((((((((Ultrasound[Title/Abstract]) OR Ultrasonograph[Title/Abstract]) OR Ultrasound Imaging[Title/Abstract]) OR Ultrasound Imagings[Title/Abstract]) OR Imaging, Ultrasound[Title/Abstract]) OR Diagnostic Ultrasound[Title/Abstract]) OR Ultrasound, Diagnostic[Title/Abstract]) OR Medical Sonography [Title/Abstract]) OR Sonography, Medical[Title/Abstract]) OR Echography[Title/Abstract]) OR Echotomography[Title/Abstract]) OR Diagnosis, Ultrasonic[Title/Abstract]) OR Ultrasonic Tomography[Title/Abstract]) OR “Ultrasonography”[Mesh])) AND (((((diagnose[Title/Abstract]) OR diagnosis[Title/Abstract]) OR screening[Title/Abstract]) OR diagnostic[Title/Abstract])). Studies published in Chinese were searched in CNKI and VIP by using the corresponding Chinese terms.

### 2.2. Inclusion and Exclusion Criteria

The final included studies met the following inclusion criteria: (1) evaluation of the diagnostic potential of HSF for BA, (2) case-control design with a control group of patients with non-BA disease, and (3) sufficient data to calculate the diagnostic parameters. Exclusion criteria were as follows: (1) duplicate publications; (2) letters, editorials, and case reports or reviews; and (3) studies lacking complete data.

### 2.3. Data Extraction and Quality Assessment

Two reviewers independently extracted the following necessary data: (1) name of the first author, (2) year of publication, (3) country of study, (4) ethnicity, (5) number of patients and mean age of patients in the case and control groups, and (6) the diagnostic outcomes including sensitivity and specificity. In addition, Quality Assessment of Diagnostic Accuracy Studies 2 (QUADAS-2) was applied to assess the quality of the included studies in four key domains (patient selection, index test, reference standard, and flow and timing) [24].

### 2.4. Statistical Analysis

The bivariate meta-analysis model was employed in our analysis to calculate the diagnostic parameters, including positive likelihood ratio (PLR), negative likelihood ratio (NLR), diagnostic odds ratio (DOR), and their corresponding 95% confidence intervals (95% CI) [25]. The summary receiver operating characteristic (SROC) curve was plotted based on the sensitivity and specificity of each study to evaluate the accuracy of HSF in the diagnosis of BA. Additionally, heterogeneity was calculated by the *Q* test and *I*^2^ test. *P* < 0.05 for the *Q* test or *I*^2^ > 50% indicated significant heterogeneity, and under these circumstances, the random-effects model was employed [26]. Furthermore, subgroup analysis and metaregression analysis were performed to identify the potential sources of heterogeneity. Subgroup analysis was performed by using the following covariates: (1) mean age < 90 days versus mean age≧90 days, (2) study design (prospective versus retrospective), (3) cases (≤60 versus >60), and (4) the final diagnosis method (intraoperative cholangiography or surgery or histology versus unclear). Finally, Begg's test was employed to assess whether there was any publication bias in the included studies, where *P* < 0.10 indicated a significant publication bias [27]. All statistical analyses were conducted using the STATA 12.0 and RevMan 5.2 software.

## 3. Results

### 3.1. Literature Search

**Figure 1:**
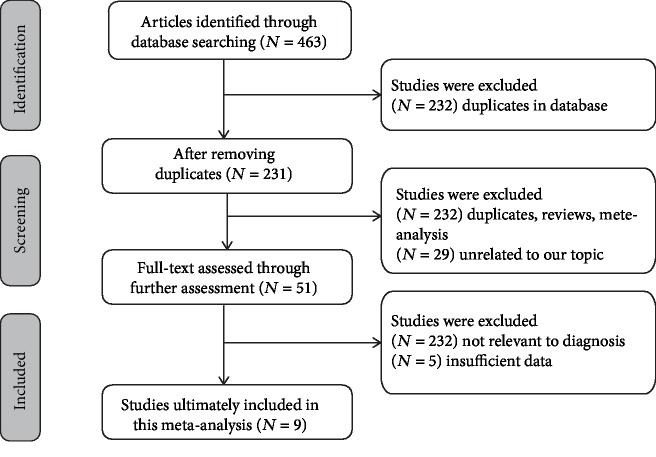
Flow chart of literature search and study selection. Nine case-control studies were included in this meta-analysis.

The initial literature search identified a total of 463 published records from PubMed, EMBASE, CNKI, and VIP. From these records, 232 were excluded as duplicate publications. Then, 231 articles were left behind for the next assessment. Using prudent judgment, 180 articles were excluded as they were reviews and letters or were not related to the theme of BA or HSF. Thus, 51 articles were available for further examination. By reviewing the full text of the remaining articles, 42 articles having insufficient data or no relevance to the diagnosis were rejected. Finally, nine studies related to HSF in the early detection of BA were included in this meta-analysis. The flow diagram of the literature search process is presented in Figure 1.

### 3.2. Study Characteristics

The main characteristics of the nine studies from eight articles are summarized in Table 1. Nine studies involving 368 patients and 469 healthy people from 2009 to 2017 were included in this meta-analysis. Studies most commonly originated from China (4/9 studies), followed by South Korea (3/9 studies) and Egypt (2/9 studies). Among these nine studies, two studies were conducted before the year 2010. In seven studies, the diagnosis was confirmed by surgery or biopsy, while the other two studies did not mention how the final diagnosis of BA was made. Besides, the mean age of patients in six studies was <90 days, and the mean age of patients in the other three studies was more than 90 days. The QUADAS-2 system was employed to assess the quality of included articles (Table 2).

### 3.3. Diagnostic Accuracy of HSF in BA

**Figure 2:**
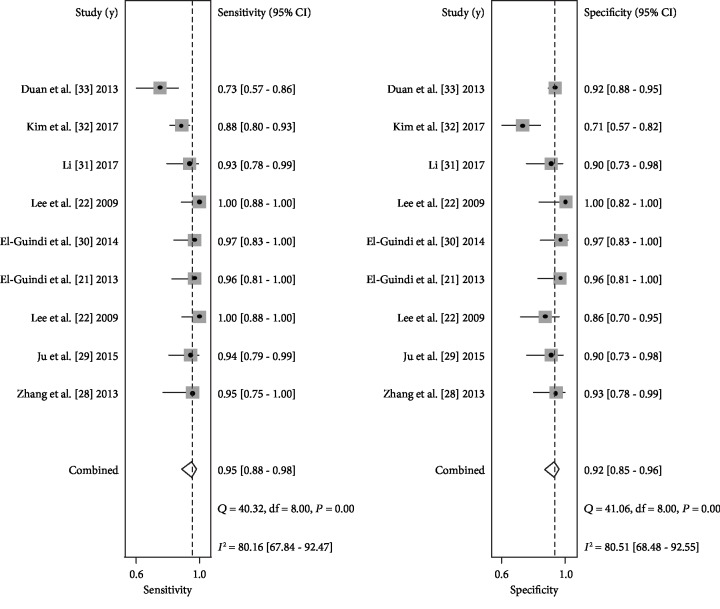
Forest plots of pooled sensitivity and specificity for HSF in the diagnosis of BA.

**Figure 3:**
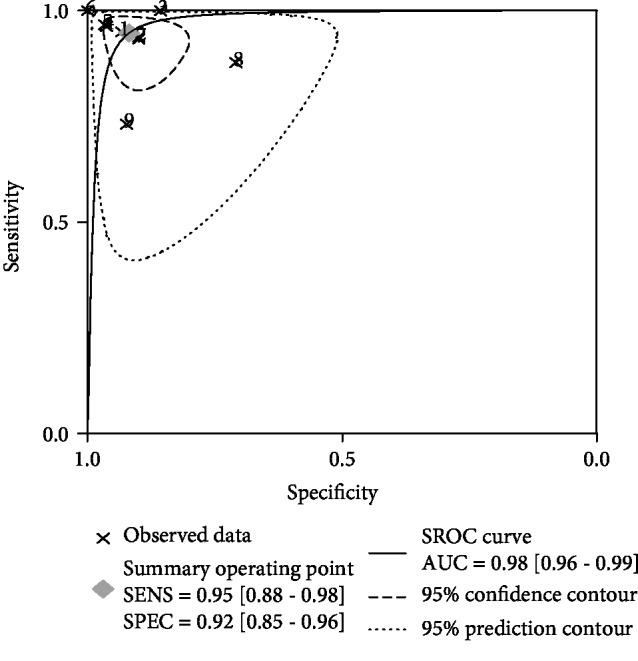
Summary receiver operating characteristic (SROC) curve for HSF in the diagnosis of BA.

Forest plots of pooled data from nine studies, concerning the sensitivity and specificity of HSF in diagnosing BA, are shown in Figure 2. Since significant heterogeneity was observed among the included studies, we choose the random-effects model in the subsequent analysis to validate the accuracy of HSF. The overall diagnostic results were as follows: sensitivity, 0.95 (95% CI 0.88-0.98); specificity, 0.92 (95% CI 0.85-0.96); PLR, 11.6 (95% CI 6.3-21.5); NLR, 0.06 (95% CI 0.02-0.14); and DOR, 201 (95% CI 59-689). In addition, the SROC curve was calculated and plotted in Figure 3, with an AUC of 0.98 (95% CI 0.96-0.99), indicating a relatively high diagnostic accuracy.

### 3.4. Metaregression and Subgroup Analysis

Considering that significant heterogeneity existed in this meta-analysis, we performed metaregression and subgroup analysis to identify the potential sources of heterogeneity. Based on the preidentified variables, such as number of cases and controls, mean age and study design, and difference in the gold standard, the result of metaregression is shown in Table 3, and it indicated that the study with mean age≧90 days caused heterogeneity (*P* < 0.05). We then conducted the subgroup analysis based on these covariates. After removing the studies with mean age≧90 days, the diagnostic value of the remaining studies showed a sensitivity of 0.92 (95% CI 0.87-0.95) and specificity of 0.93 (95% CI 0.89-0.95) (Table 3). Compared with the overall studies, we found that the diagnostic value of the subgroup did not improve significantly, thus indicating that our results concerning the diagnostic value of HSF for BA were relatively credible and stable despite the heterogeneity.

**Figure 4:**
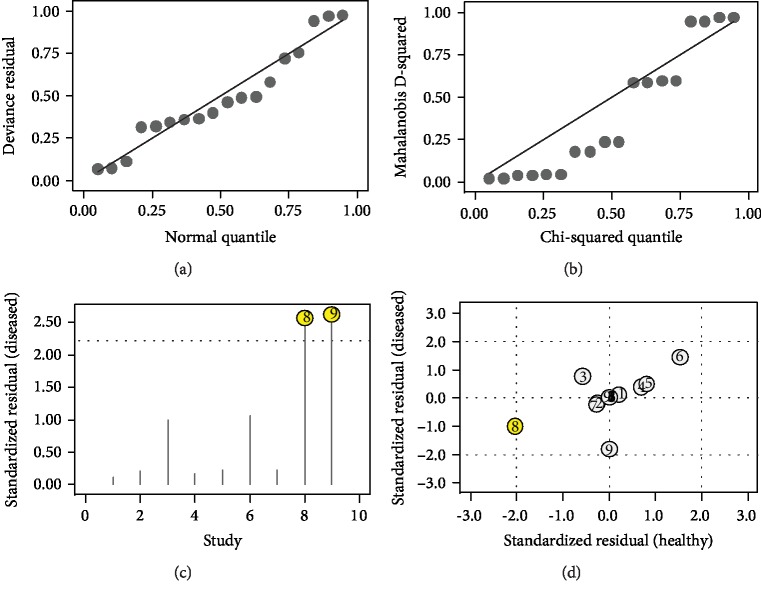
Influence analysis and outlier detection: (a) goodness of fit, (b) bivariate normality, (c) influence analysis, and (d) outlier detection.

We demonstrated that the bivariate random-effects model was robust in this meta-analysis by the goodness of fit and bivariate normality analyses (Figure 4). Influence analysis and outlier detection were then used to identify whether there were any outliers in all studies. After one outlier was identified and excluded, we conducted the same analysis for the remaining studies. Compared with the previous results, the sensitivity decreased from 0.95 to 0.92, the PLR dropped from 11.60 to 9.88, the NLR showed no change from 0.06 to 0.06, DOR decreased from 201 to 187, and the specificity remained unchanged, thus suggesting that there was no significant change from the overall analysis.

### 3.5. Publication Bias

**Figure 5:**
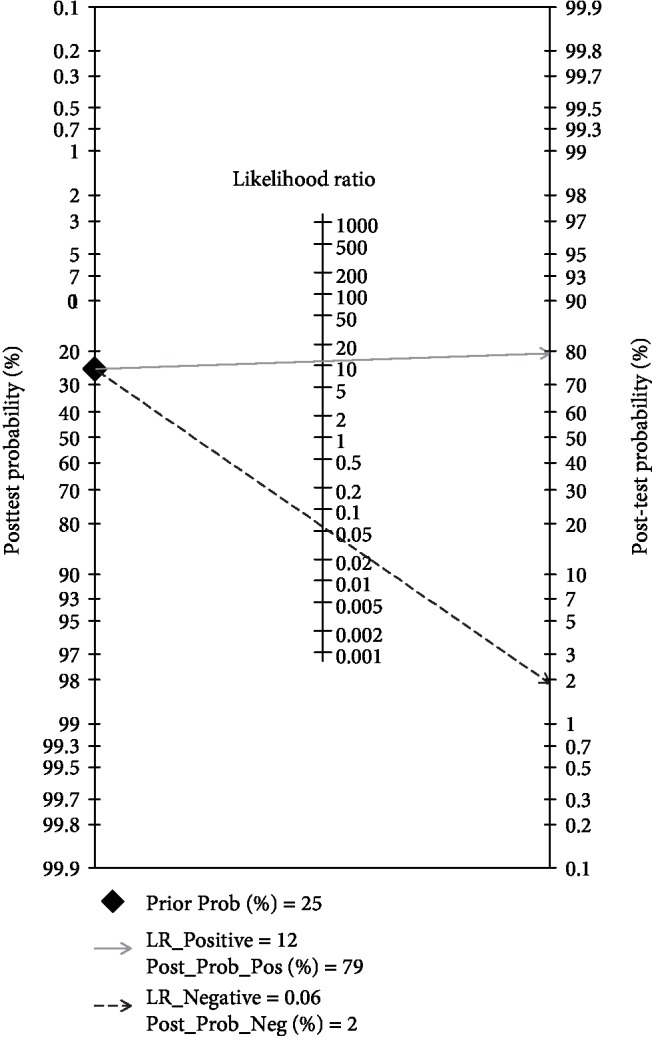
Fagan's nomogram in assessment of the test probabilities after HSF assay.

**Figure 6:**
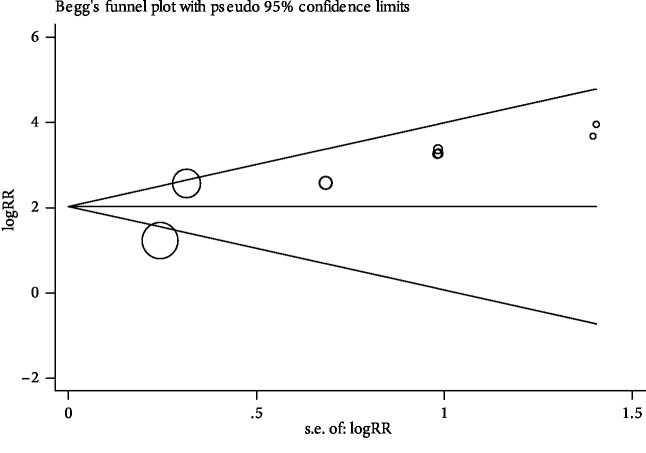
Begg's funnel plot of publication bias (*P* = 0.25).

Fagan's nomogram was used to identify the correlation between HSF and the probability of BA (Figure 5). We derived the following conclusions: there will be a pretest probability of 25% for anyone to have BA after testing the HSF; however, if a positive result is obtained, the posttest probability of having BA will increase to 79%. In contrast, if a negative result is obtained, the posttest probability of having BA will drop to 2%. Therefore, HSF as a beneficial marker would be a significant test for the diagnosis of BA. In addition, we performed Begg's test to evaluate publication bias, and we found that there was no significant publication bias in this meta-analysis (*P* value = 0.25, Figure 6).

## 4. Discussion

BA is a progressive disease condition. It presents with persistent jaundice, pale stools, and dark urine in an otherwise healthy infant, and its incidence in the Asia-Pacific region is higher than that elsewhere. The Kasai operation is the currently recommended first-line treatment for BA, and the earlier the Kasai operation is performed, the higher is its chance of being successful [34, 35]. Consequently, determining a reliable method for the early diagnosis of BA is very crucial as the timing of surgery correlates with the outcome.

Nowadays, ultrasonography is a key diagnostic test on the basis of several parameters, such as the presence of the triangular cord (TC) sign and an abnormal gallbladder (GB) on US (GB length and GB contractility) [36–38]. We have mentioned that the accuracy of these parameters in the diagnosis of BA is not satisfactory. According to Kim et al.'s study [39], the TC sign has high specificity (62%) but low sensitivity (100%). In El-Guindi et al.'s study [21], it was reported that a GB length of less than 20.5 mm is 81.4% sensitive and 70.3% specific for BA. Similarly, Takamizawa et al. [40] reported that GB contractility had 78% sensitivity and 72% specificity in discriminating BA. Conclusively, these parameters may not be ideal markers for the diagnosis of BA, but they would be worthy supplemental methods when combined with clinical features and techniques such as physical examination and CT/MRI scanning.

HSF, which occurs as a result of hyperplastic and hypertrophic changes in branches of the hepatic artery, could be observed in the stage of disease when bridging fibrosis leads to features of overt biliary cirrhosis [41]. Recent studies have confirmed HSF could be a more suitable clinical marker with a higher capacity for discriminating BA patients from non-BA subjects. Lee et al. [22] performed a study in 18 patients suspected of having BA and 15 controls, and they found that HSF was 100% sensitive and 86% specific in distinguishing between BA and non-BA (AUC 0.995). Similarly, El-Guindi et al.'s report [21] revealed that the sensitivity and specificity of HSF were 96% and 96%, respectively, and it concluded that HSF could be a single parameter in the diagnosis of BA with better performance. However, a lack of systematic evaluation weakens this conclusion. Therefore, we carried out this comprehensive research in a clinical context to precisely evaluate the diagnostic accuracy of HSF.

In this systematic review, nine diagnostic studies were included to investigate whether HSF is a useful parameter for diagnosing BA. We demonstrated that HSF had excellent diagnostic accuracy and yielded a combined AUC of 0.98 (95% CI 0.96-0.99), with pooled sensitivity of 0.95 (95% CI 0.88-0.98) and pooled specificity of 0.92 (95% CI 0.85-0.96) for discriminating BA cases in the diagnosis of BA. The PLR and NLR were used to estimate the diagnostic accuracy at a clinical level. The pooled PLR of 11.6 suggested that BA patients have an approximately 11.6-fold higher chance of HSF positivity compared with non-BA controls. The pooled NLR of 0.06 indicated that the possibility of individuals having BA was 6% if the HSF was negative. Furthermore, the DOR value of 201 attracted our attention and indicated that if someone tests positive, then he/she has a 201-fold higher chance of having BA than the subject who tests negative.

Metaregression and subgroup analysis were performed to analyze the potential sources of heterogeneity. Based on the *I*^2^ test, the random-effects model was selected. According to the major attributes of primary studies, heterogeneity was influenced by the mean age (*P* = 0.04), rather than by the sample size (*P* = 0.16), final diagnosis (*P* = 0.30), and study design (*P* = 0.07). If we removed the study with mean age≧90 days and then calculated the parameters of diagnostic accuracy of the remaining seven studies, new results were noted, and they showed no significant difference compared with the overall pooled results. It can be concluded that these studies have a little effect on the whole diagnostic accuracy (Table 2). As mentioned above, early diagnosis of BA exerts a significant effect on this disease. Through subgroup analysis, we noted that the diagnostic accuracy of HSF for age < 90 days is higher when compared with that for age ≥ 90 days, which indicated that it may be the ideal signature in early detection of BA. Other covariates, such as sample size, difference in gold standard, and study design, were confirmed to show no significant difference from the overall pooled results.

Additionally, we concluded from the goodness of fit and bivariate normality analyses (Figures 6(a) and 6(b)) that the bivariate model was moderately robust. Influence analysis and outlier detection identified one outlier (Figures 6(c) and 6(d)). After removing the outlier and performing the same analyses for the remaining studies, we found that the overall parameters of diagnostic accuracy did not change significantly. Finally, Begg's funnel plot was created to investigate the publication bias of the studies. It indicated that the *P* value was 0.25, suggesting that no publication bias was present in our study.

However, several limitations to our study need to be addressed. First, sample sizes in some of the included publications were relatively small, and therefore, more studies with high quality and large study population are needed to further confirm our conclusion. Second, inconsistent results from the included studies could be attributed to technological and staffing limitations; however, sufficient data were not available to evaluate these parameters. Third, all of the studies included in this meta-analysis were performed in Asian populations, which may have led to a population selection bias. Finally, lack of access to obtain the original data from the included studies resulted in limiting our ability to perform the meta-analysis. Thus, sufficient data should be collected and analyzed in the future.

In conclusion, the results of this meta-analysis indicated that HSF provides high accuracy in distinguishing BA patients from non-BA controls in a noninvasive and highly efficient manner. It reminds us that we can deeply assess the clinical application of HSF in the early diagnosis of BA. Next, we can further standardize the diagnostic criteria for HSF and encourage more clinicians to apply HSF in the clinical diagnosis of BA. However, further studies need to be conducted to efficiently apply these findings for clinical detection of BA.

## Figures and Tables

**Table 1:** Main characteristics of the diagnostic studies included in the meta-analysis.

Author	Year	Country	Case	Control	Sensitivity	Specificity	TP	FP	FN	TN	Design
Zhang et al. [28]	2013	China	20	30	95.00%	93.33%	19	2	1	28	Respective
Ju et al. [29]	2015	China	32	30	93.75%	90.00%	30	3	2	27	Respective
Lee et al. [22]	2009	Korea	29	35	100%	85.71%	29	5	0	30	Respective
El-Guindi et al. [21]	2013	Egypt	27	27	96.30%	96.30%	26	1	1	26	Prospective
El-Guindi et al. [30]	2014	Egypt	30	30	96.67%	96.67%	29	1	1	29	Prospective
Lee et al. [22]	2009	Korea	29	19	100%	100%	29	0	0	19	Respective
Li [31]	2017	China	30	30	93.33%	90.00%	28	3	2	27	Prospective
Kim et al. [32]	2017	Korea	106	55	87.74%	70.90%	93	16	13	39	Respective
Duan et al. [33]	2013	China	65	213	73.17%	92.37%	30	18	11	218	Respective

Abbreviations: TP: true positive; FP: false positive; FN: false negative; TN: true negative.

**Table 2:** Risk of bias assessed by QUADAS-2.

Author	Risk of bias				Applicability concerns		
	Patient selection	Index test	Reference standard	Flow and timing	Patient selection	Index test	Reference standard
Lee et al. [22]	High	Low	Low	Low	Low	Low	Low
El-Guindi et al. [21]	High	Low	Low	Low	Low	Low	Low
Kim et al. [32]	High	Low	Low	Low	Low	Low	Low
El-Guindi et al. [30]	High	Low	Low	Low	Low	Low	Low
Li [31]	High	High	Low	Low	Low	Low	Low
Zhang et al. [28]	High	High	Low	Low	Low	Low	Low
Duan et al. [33]	High	Low	Low	Low	Low	Low	Low
Ju et al. [29]	High	High	Low	Low	Low	Low	Low
Lee et al. [22]	High	Low	Low	Low	Low	Low	Low

**Table 3:** Multivariate metaregression analysis for the associations of HSF with susceptibility to BA. Results of subgroup and metaregression analysis in the diagnosis meta-analysis.

	Sensitivity	95% CI		Specificity	95% CI		Regression
Design							0.07
Respective	0.9	0.85	0.93	0.89	0.86	0.92	
Prospective	0.95	0.89	0.99	0.94	0.87	0.98	
Mean age							0.04
<90	0.92	0.87	0.95	0.93	0.89	0.95	
≧90	0.90	0.84	0.94	0.82	0.74	0.88	
Sample size							0.16
>60	0.89	0.84	0.93	0.89	0.85	0.92	
≤60	0.95	0.89	0.99	0.94	0.88	0.98	
Final diagnose							0.30
Surgery/biopsy	0.89	0.83	0.94	0.93	0.9	0.95	
Unclear	0.89	0.83	0.94	0.78	0.67	0.86	

Abbreviation: CI: confidence interval.
